# Hydrodynamics Regulate Longitudinal Plankton Community Structure in an Alpine Cascade Reservoir System

**DOI:** 10.3389/fmicb.2021.749888

**Published:** 2021-10-27

**Authors:** Yang Liu, Chengyan Li, Shenglong Jian, Shiyu Miao, Kemao Li, Hongtao Guan, Yaqi Mao, Zhongyi Wang, Changzhong Li

**Affiliations:** ^1^College of Eco-Environmental Engineering, Qinghai University, Xining, China; ^2^State Key Laboratory of Plateau Ecology and Agriculture, Qinghai University, Xining, China; ^3^Qinghai Provincial Fishery Environmental Monitoring Center, Xining, China; ^4^The Key Laboratory of Plateau Aquatic Organism and Ecological Environment in Qinghai, Qinghai Provincial Fishery Environmental Monitoring Center, Xining, China

**Keywords:** phytoplankton, zooplankton, cascade reservoirs, community pattern, damming, Qinghai-Tibetan plateau

## Abstract

Previous studies report significant changes on biotic communities caused by cascade reservoir construction. However, factors regulating the spatial–temporal plankton patterns in alpine cascade reservoir systems have not been fully explored. The current study explored effects of environmental factors on the longitudinal plankton patterns, through a 5-year-long study on the environmental factors and communities of phytoplankton and zooplankton in an alpine cascade reservoir system located upstream of Yellow River region. The findings showed that phytoplankton and zooplankton species numbers in the studied cascade reservoir system were mainly regulated by the hydrological regime, whereas nutrient conditions did not significantly affect the number of species. Abundance and biovolume of phytoplankton in cascade reservoirs were modulated by the hydrological regime and nutrient conditions. The drainage rate, N:P ratio, and sediment content in cascade reservoirs were negatively correlated with abundance and biovolume of phytoplankton. Abundance and biovolume of zooplankton were not significantly correlated with the hydrological regime but showed a strong positive correlation with nutrient conditions in cascade reservoirs. Shannon–Wiener index (*H’*) and the Pielou index (*J*) of phytoplankton were mainly regulated by the hydrological regime factors, such as drainage rate and sediment content in cascade reservoirs. However, temperature and nutrient conditions were the main factors that regulated the Shannon–Wiener index (*H’*) and the Pielou index (*J*) of zooplankton. Species number, abundance, and biovolume of phytoplankton showed a significant positive correlation with those of zooplankton. Hydrodynamics and nutrient conditions contributed differently in regulating community structure of phytoplankton or zooplankton. These findings provide an understanding of factors that modulate longitudinal plankton community patterns in cascade reservoir systems.

## Introduction

Several dams are built on rivers worldwide to increase the use of water resources and meet surging domestic power demand (Graf, 1999; Finer and Jenkins, 2012; Grumbine and Pandit, 2013). Dams cause longitudinal river discontinuity and convert some original river ecosystems into reservoir or lake ecosystems with low water flow, high water transparency, and raised temperature (Magilligan and Nislow, 2005; Okuku et al., 2016; Van Cappellen and Maavara, 2016). Serial constructions of dams lead to a cascade reservoir system along the river. Several studies have explored the effects of cascade reservoirs on river ecosystems (Fan et al., 2015; Algarte et al., 2016; Wang Y. et al., 2016; Kang et al., 2017; Baumgartner et al., 2020). Studies report that cascade reservoirs cause significant changes in phytoplankton (Padisák et al., 2000; Silva et al., 2005; Wang et al., 2018), zooplankton (Hart, 2004; Perbiche-Neves and Nogueira, 2010; Okuku et al., 2016), benthic animals (Callisto et al., 2005), fish (Draštík et al., 2008), water physiochemical characteristics (Jorcin and Nogueira, 2005; Karnaukhova, 2007), and hydrological regimes (Matsuno et al., 2003) in river ecosystems. The effect of the construction of cascade reservoirs on rivers is significant for rivers in high-altitude areas whose ecological environment is more fragile. However, studies on the effects of construction of cascade reservoirs on alpine river ecosystems are limited.

Plankton, including phytoplankton and zooplankton, plays an important role in material circulation and energy flow in river or aquatic ecosystems (Cury et al., 2000; Richardson and Schoeman, 2004; Frederiksen et al., 2006). Phytoplankton is the main primary producer, whereas zooplankton is a group connecting the food webs of river ecosystems and are the most sensitive species to changes (Padisák et al., 2000; Silva et al., 2005; Perbiche-Neves and Nogueira, 2010; Wang et al., 2018). Therefore, community characteristics and dynamics of plankton are often used to explore the structure and functioning of river ecosystems, help in identifying regulating factors of plankton dynamics, and in evaluating effects of human activities on natural river ecosystems (Paerl et al., 2010; Okuku et al., 2016).

Previous studies on phytoplankton or zooplankton in individual reservoir reported that hydrology, nutrient availability, and biota interactions were the main controlling factors of plankton composition and biomass in reservoirs (Rangel et al., 2012; Beaver et al., 2013; Chang et al., 2014; Silva et al., 2014; Amaral et al., 2020). However, the detailed relationship between plankton community and these environmental factors was reservoir specific and more complex since conflicting results were reported (Beaver et al., 2013; Silva et al., 2014). Previous studies on the plankton pattern formation mechanism in reservoirs that omitted either phytoplankton or zooplankton may be inaccurate owing to the predation relationship between phytoplankton and zooplankton (Chang et al., 2014). Lack of a detailed investigation on environmental variables, phytoplankton, zooplankton, and fish characteristics may partly account for the contradicting findings from previous studies (Chang et al., 2014). Although studies on phytoplankton or zooplankton community in individual reservoirs are abundant, studies on longitudinal plankton community pattern and corresponding structuring factors after cascade reservoir construction are few (Okuku et al., 2016). Most studies only explored the effects on either phytoplankton or zooplankton (Padisák et al., 2000; Hart, 2004; Silva et al., 2005; Perbiche-Neves and Nogueira, 2010; Wang et al., 2018), and studies that explored both phytoplankton and zooplankton in a continuous cascade reservoir system are limited (Silva et al., 2005; Okuku et al., 2016; Wang et al., 2018). Environmental variables, phytoplankton community characteristics, zooplankton community characteristics, and fish community characteristics should be explored together to avoid potentially conflicting conclusions among studies. However, systematic and longtime span study of both phytoplankton community and zooplankton community in cascade reservoir system have not been conducted. Longitudinal plankton community pattern and corresponding regulating factors in cascade reservoir systems are not fully understood.

In the current study, the longitudinal distribution pattern of plankton and the corresponding regulation factors were explored for the first time in a high-altitude alpine cascade reservoir system. Longitudinal variation and dynamics of both phytoplankton and zooplankton community characteristics, including species number/composition, abundance, biovolume and biodiversity index, fish community composition, and environmental factors (environmental variables–phytoplankton–zooplankton–fish) were sampled for 5 years in the 300-km alpine and oligotrophic plateau cascade reservoirs. This study sought to (1) describe the longitudinal variation of plankton community pattern along the alpine cascade reservoirs, (2) promote understanding of environmental variables–phytoplankton–zooplankton–fish interactions in cascade reservoir systems, and (3) identify factors regulating plankton community pattern in cascade reservoir systems. The findings of the current study will promote understanding of factors that modulate the longitudinal plankton community patterns in cascade reservoir systems worldwide.

## Materials and Methods

### Study Area

Yellow River is the second largest river in China and the sixth largest in the world. The altitude upstream of Yellow River varies between 2,000 and 4,000 m above sea level. Twelve reservoirs have been built between Longyang Gorge (LYG) and Jishi Gorge (JSG) upstream of Yellow River, forming the largest cascade reservoir system in China. The cascade reservoir system is approximately 300-km long and 800-m drop, and has an average annual runoff of 2.8 × 10^10^ m^3^. All reservoirs are weekly or daily regulated reservoirs except for the upstream LYG, which is an annually regulated reservoir (Miao et al., 2020). Detailed morphological characteristics of each cascade reservoir have been reported by Miao et al. (2020). The cascade reservoir system is oligotrophic with a low TP and a high N:P ratio (Miao et al., 2020). Furthermore, it comprises a broad latitudinal gradient of depth, water residence time, and nutrient levels. The drainage rate was calculated by dividing individual reservoir storage by the daily outflow.

### Sample Collection and Analysis

**FIGURE 1:**
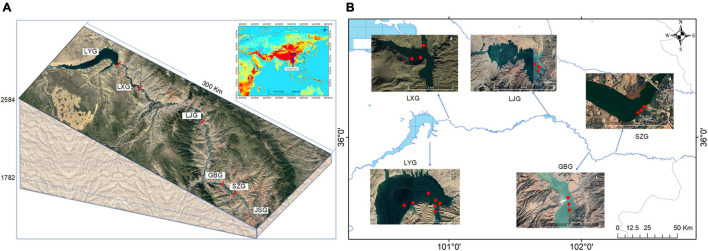
Schematic diagram of the location of the cascade reservoirs **(A)** and sampling points in the cascade reservoirs **(B)**. Dams are shown by solid bars.

To explore the plankton community pattern and corresponding regulating factors after cascade reservoir construction, sampling sites were established in the lacustrine zone of each reservoir in Longyang Gorge (LYG), Laxi Gorge (LXG), Lijia Gorge (LJG), Gongbo Gorge (GBG), and Suzhi Gorge (SZG) (Figure 1). Sampling was carried out four times yearly from March to October for 5 years from 2013 to 2017.

Sample collection, species identification, and biovolume determination of plankton were performed following the Specification for Freshwater Plankton Surveys (SC/T 9402-2010). In the current study, qualitative and quantitative samples of both phytoplankton and zooplankton were collected. Species identification and counting were performed using an optical microscope with a phytoplankton counting chamber. Plankton biovolume (wet weight) was estimated using biovolume (mm^3^ L^–1^) and converted from the plankton abundance using quantitative plankton samples (Hillebrand et al., 1999; Sun and Liu, 2003). Fishes were caught with approval by the local authority using cages and gill net methods (SC/T 9102.1-2007) to evaluate predation pressure on zooplankton. Primary species identification and numbering was conducted in the field. Fishes were released back after identification, photographing, and numbering.

GPS coordinates, altitude, water temperature, and pH of the sampling points were determined on-site. Dissolved oxygen (DO) level was determined using the iodometric method (GB 7489-87) within 24 h after sampling. Total nitrogen (TN) was determined by ultraviolet spectrophotometry after digestion of samples with alkaline potassium persulfate (HJ 636-2012). Total phosphorus (TP) was determined using ammonium molybdate spectrophotometry method (GB/T 1183-1989). Chemical oxygen demand (COD_*Mn*_) was evaluated using alkaline potassium permanganate titration method (GB 11892-1989). The level of suspended solid (SS) was determined using gravimetric method (GB 11901-1989). Biological oxygen demand (BOD_5_) was determined by dilution and inoculation method (HJ 505-2009).

### Statistical Analysis

Data on plankton abundance, biomass, and biodiversity indexes, and environmental variables were presented as means ± standard deviations (mean ± SD) to show yearly variability. Phytoplankton and zooplankton community structures were expressed as species number, abundance, biomass, Shannon–Wiener diversity indexes, and Pielou evenness indexes. The 5-year results were used to show plankton community composition in the cascade reservoir system. Plankton diversity was measured by Shannon–Wiener diversity and Pielou evenness indexes.

Remote-sensing images of sampling points were generated by Google Earth (version 7.3.3.7699). Origin software (version 8.6) was used to map the longitudinal and temporal variations of plankton characteristics. Corrplot package (version 0.84) was used for Pearson’s correlation analysis between phytoplankton communities, zooplankton communities, and environmental parameters. Detrended correspondence analysis (DCA) showed that the size of the gradient was less than 3; thus, redundancy analysis (RDA) was conducted among the Hellinger transformed phytoplankton, zooplankton, and environmental factors using CANOCO 5.0. Network analysis between plankton community characteristics and environmental factors in the cascade reservoirs was performed using Cytoscape tool (Version 3.8.1). Mean values of plankton abundance, biovolume, and biodiversity index for each site over the 5 years were used for Pearson’s correlation analysis, redundancy analysis, and network analysis. The values of ^∗^*p* < 0.05, ^∗∗^*p* < 0.01, and ^∗∗∗^*p* < 0.001 showed significant correlation.

## Results

### Phytoplankton Community Composition and Longitudinal Variation

**FIGURE 2:**
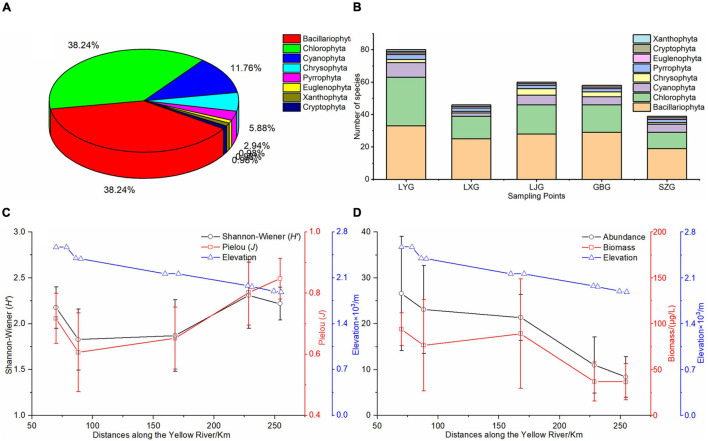
Phytoplankton pattern in the cascade reservoirs. **(A)** Phytoplankton species composition over the 5 years of study, **(B)** phytoplankton species composition in each cascade reservoir, **(C)** longitudinal changes in the Shannon–Wiener index (*H’*) and Pielou index (*J*) of phytoplankton along the cascade reservoirs, and **(D)** longitudinal changes in phytoplankton abundance and biovolume along the cascade reservoirs. Locations of cascade reservoirs are recorded as kilometers upriver from the inlet site of the LYR.

Eight phyla with 102 species of phytoplankton were identified in the five cascade reservoirs over the 5 years with 39 Bacillariophyta species (38.2%) and 39 Chlorophyta species (38.2%) (Figure 2A). The number of phytoplankton species, especially Bacillariophyta, Chlorophyta, and Cyanophyta, determined in LYG was significantly higher compared with that in the other reservoirs (Figures 2B,C). *Synedra acus*, *Navicula* sp., *Cymbella* sp., *Diatoma vulgare*, *Synedra* sp., *Cyclotella* sp., and *Achnanthes* sp. in the Bacillariophyta phylum, *Chlamydomona* sp. and *Ulothrix* sp. in Chlorophyta, and *Phormidium* sp. and *Oscillatoria tenuis* in Cyanophyta and *Ceratium hirundinella* in the Pyrrophyta phylum were the widely distributed species found in all five cascade reservoirs. The findings showed that the Bacillariophyta–Chlorophyta pattern of phytoplankton composition did not change over time. The average phytoplankton abundance and biovolume were 1.8 × 10^5^ cells/L and 66.6 μg/L, respectively, in the five cascade reservoirs over the 5 years of study. Abundance and biovolume of phytoplankton decreased longitudinally along the river from upstream LYG to downstream SZG (Figure 2C). The average phytoplankton Shannon–Wiener diversity index and Pielou index were 2.08 and 0.73, respectively, in the five cascade reservoirs over the 5 years (Figure 2D).

### Zooplankton Community Composition and Longitudinal Variation

**FIGURE 3:**
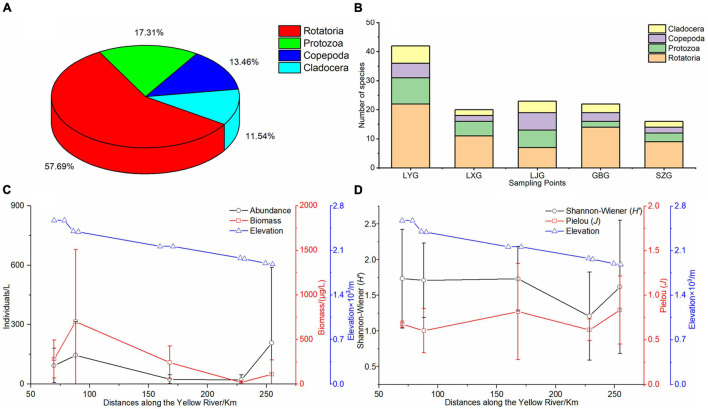
Zooplankton pattern in the cascade reservoirs. **(A)** Zooplankton species composition over the 5 years of study, **(B)** zooplankton species composition in each cascade reservoir, **(C)** longitudinal changes in zooplankton abundance and biovolume along the cascade reservoirs, and **(D)** longitudinal changes in the Shannon–Wiener index (*H’*) and Pielou index (*J*) of zooplankton along the cascade reservoirs.

Four phyla comprising 52 zooplankton species were identified in the five cascade reservoirs over the 5 years with 30 Rotatoria species (57.7%) and 9 Protozoa species (17.3%) (Figure 3A). Rotatoria and Protozoa were the dominant species in the five cascade reservoirs. Although Rotatoria and Protozoa were the predominant zooplankton species in the five cascade reservoirs, the zooplankton composition in each cascade reservoir was significantly different (Figure 3B). Analysis showed that LYG had the maximum number of zooplankton species (Figure 3B). *Acanthocystis* sp. and *Tintinnopsis wangi* in the Protozoa phylum, *Synchaeta* sp., *Keratella cochlearis*, *Polyarthra trigla*, and *Asplanchna* sp. in the Rotatoria phylum, and *Bosmina longirostris* in the Cladocera phylum were widely distributed in the reservoirs. Zooplankton species identified over the 5 years gradually decreased longitudinally from upstream LYG to downstream SZG (Figure 3B). Average zooplankton abundance, biovolume, Shannon–Wiener diversity index, and Pielou index were 294.44 cells/L, 402.1 μg/L, 1.56, and 0.73, respectively, in the five cascade reservoirs over the 5 years (Figures 3C,D).

### Factors Modulating the Longitudinal Phytoplankton Pattern

**FIGURE 4:**
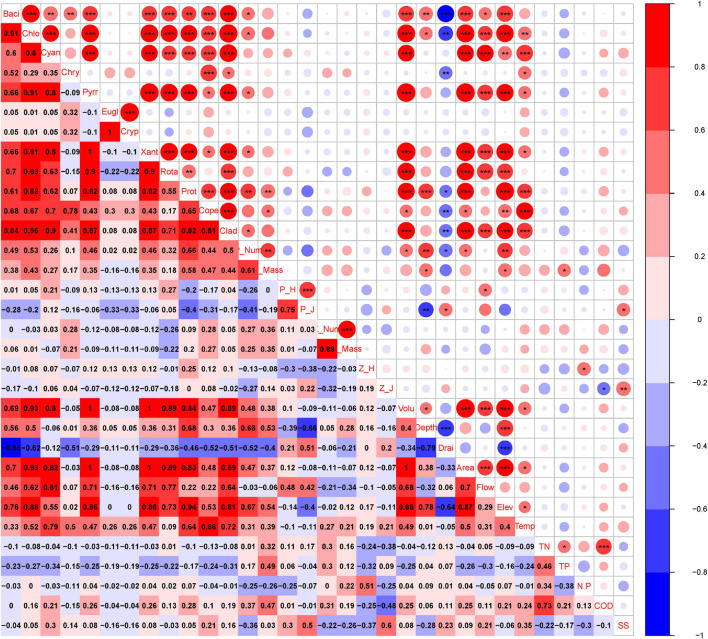
Pearson’s correlation analysis between plankton and environmental factors in the cascade reservoirs over the 5 years. Baci, Bacillariophyta; Chlo, Chlorophyta; Cyan, Cyanophyta; Chry, Chrysophyta; Pryy, Pyrrophyta; Eugl, Euglenophyta; Cryp, Cryptophyta; Xant, Xanthophyta; Rota, Rotatoria; Prot, Protozoa; Cope, Copepoda; Clad, Cladocera; P_Num, phytoplankton abundance; P_Mass, phytoplankton biovolume; P_H, phytoplankton Shannon–Wiener diversity index; P_J, phytoplankton Pielou index; Z_Num, zooplankton abundance; Z_Mass, zooplankton biovolume; Z_H, zooplankton Shannon–Wiener diversity index; Z_J, zooplankton Pielou index; Volu, volume; depth, depth; Drai, drainage rate; Area, area; Flow, runoff; Elev, altitude; Temp, temperature; TN, total nitrogen; TP, total phosphorus; N.P, N:P ratio; COD, chemical oxygen demand; SS, suspended substances. *, **, and *** in the upper part indicates significance. Numbers in the lower part indicate correlation coefficient.

**FIGURE 5:**
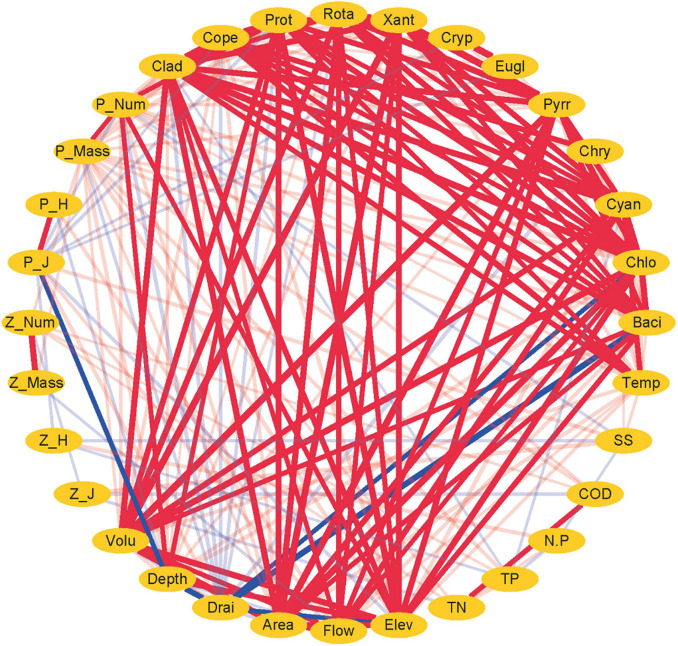
Network analysis between plankton and environmental factors in the cascade reservoirs over the 5 years. Red lines indicate positive correlation. Blue lines indicate negative correlation. Larger width of lines indicates a stronger correlation.

**FIGURE 6:**
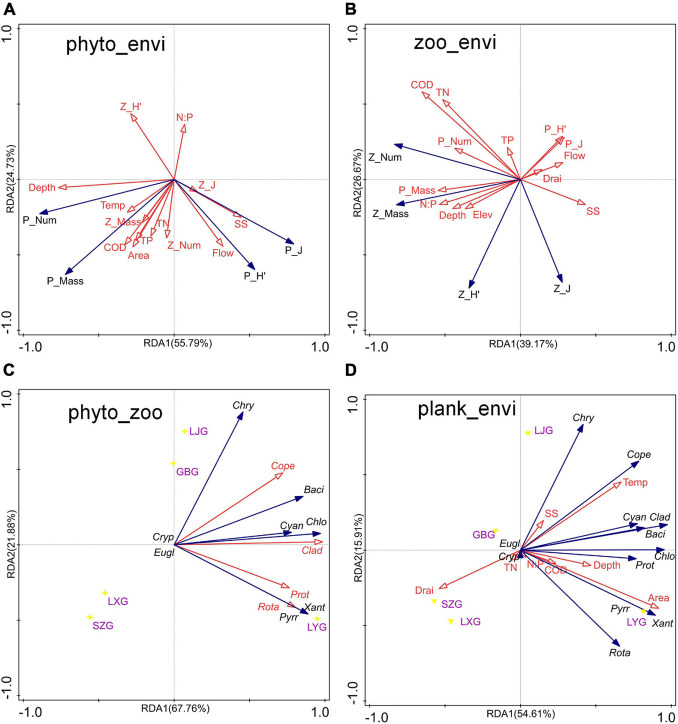
Redundancy analysis (RDA) among phytoplankton, zooplankton, and environmental factors in the cascade reservoirs over the 5 years. **(A)** RDA of phytoplankton community composition and environmental factors, **(B)** RDA of zooplankton community composition and environmental factors, **(C)** RDA of the number of phytoplankton and zooplankton species, and **(D)** RDA of the number of plankton species and environmental factors.

The findings showed that variations in phytoplankton composition among cascade reservoirs were mainly shaped by hydrological regime; however, water chemistry modulated phytoplankton composition (Figures 4–6). The number of phytoplankton species was mainly modulated by the hydrological regime of cascade reservoirs (Figures 4, 5). Notably, volume, area, flow, drainage rate, and altitude showed significant effects on modulation of phytoplankton species (Figures 4–6). The drainage rate of cascade reservoirs showed significant negative effects on phytoplankton species number (Figures 4–6). A higher drainage rate was correlated with lower phytoplankton species number in cascade reservoirs (Figures 4, 5). Decrease in drainage rate significantly increased the species number of Bacillariophyta and Chlorophyta in the alpine and oligotrophic cascade reservoirs in the upstream Yellow River (Figures 4, 5). Nutrient parameters, such as COD, TN, and TP levels, were not significantly correlated with phytoplankton species number in the studied cascade reservoirs (Figures 4, 5). However, abundance and biovolume of phytoplankton were synergistically affected by hydrological regime and nutrient levels (Figures 4–6). Drainage rate, N:P ratio, and sediment content were negatively correlated with abundance and biovolume of phytoplankton (Figures 4, 5). This finding showed that phytoplankton abundance and biovolume decreased with increase in drainage rate and N:P ratio in cascade reservoirs. The Shannon–Wiener index (*H’*) and the Pielou index (*J*) of phytoplankton were modulated by water depth, drainage rate, flow and sediment content (Figures 4, 5). Notably, abundance and biovolume of phytoplankton were low, whereas, the Shannon–Wiener index (*H’*) and Pielou index (*J*) of phytoplankton were high in cascade reservoirs with high sediment content and high drainage rate (Figures 4, 5). The findings showed high abundance and biovolume of phytoplankton in deep cascade reservoirs, whereas the Shannon–Wiener index (H’) and the Pielou index (*J*) of phytoplankton (P_H’ and P_J) were low (Figures 4, 5). These findings show that water depth, drainage rate, and sediment content played an important role in modulating phytoplankton community composition in cascade reservoir systems.

### Factors Modulating the Longitudinal Zooplankton Pattern

Although Rotatoria and Protozoa were the most dominant species in all cascade reservoirs, the species number, abundance, biovolume, and biodiversity index of zooplankton differed along cascade reservoirs (Figure 3B). The species number of zooplankton was mainly modulated by hydrological regime in the studied cascade reservoir system (Figures 4–6). Notably, volume, area, flow, and drainage rate significantly modulated the number of zooplankton species (Figures 4, 5). The drainage rate showed significant negative effects on the species number of zooplankton (Figure 4). A high drainage rate was correlated with lower zooplankton species number (Figures 4, 5). Nutrients, such as COD, TN, and TP, showed insignificant effects on zooplankton species number (Figures 4, 5). Notably, abundance and biovolume of zooplankton was not significantly correlated with the hydrological regime. However, a strong positive correlation with temperature, TN, TP, and COD was observed, whereas abundance and biovolume of zooplankton were negatively correlated with the sediment content (Figure 4). These findings indicated that abundance and biovolume of zooplankton were higher in cascade reservoirs with high water temperature, high nutrient levels, and low sediment content. Contrary to that of phytoplankton, the biodiversity of zooplankton was not significantly correlated with the hydrological regime; however, it showed a significant correlation with temperature and nutrient levels (Figures 4, 5). Regulation of biodiversity of zooplankton by temperature and nutrient levels was probably through regulation of abundance and biovolume of phytoplankton. Sediment content showed different effects on biodiversity of zooplankton compared with that of phytoplankton (Figures 4–6). Sediment content in cascade reservoirs was correlated with a low Shannon–Wiener index (*H’*), and a high Pielou index (*J*) of zooplankton (Figure 4). These findings show the important role of drainage rate, temperature, nutrient levels, and sediment content in regulating community pattern of zooplankton in cascade reservoir systems.

### Correlation Analysis Between Phytoplankton and Zooplankton Communities

Pearson’s correlation analysis (Figure 4) and network analysis (Figure 5) showed that the phytoplankton species number was positively correlated with zooplankton species number in the cascade reservoirs over the 5 years (Figures 4, 5). Correlation coefficients between Chlorophyta and the four zooplankton phyla were more than 0.9 (Figures 4, 5). Furthermore, abundance and biovolume of phytoplankton showed a positive correlation with abundance and biovolume of zooplankton (Figures 4, 5). This finding is consistent with the predatory relationship between phytoplankton and zooplankton. The Shannon–Wiener index of phytoplankton was negatively correlated with the Shannon–Wiener index of zooplankton in the cascade reservoirs (Figures 4, 5). Abundance and biovolume were negatively correlated with biodiversity for both phytoplankton and zooplankton.

## Discussion

### Phytoplankton Community Variation Regulated by Physicochemical Factors

This study explored the effects of different factors on the plankton community patterns in an alpine and oligotrophic cascade reservoir system. Plankton samples were collected for 5 years in the cascade reservoirs, to ensure that the study data were systematic for reliable conclusions. Phytoplankton species composition in the alpine cascade reservoirs showed a dominant Bacillariophyta–Chlorophyta pattern (Qiu et al., 2019). Bacillariophyta is dominant at low temperature (Schabhüttl et al., 2013) and high turbulence (Harris and Baxter, 1996) riverine waters (Reynolds et al., 2002; Beaver et al., 2013). The cascade reservoirs in the current study are located in the upstream Yellow River in the alpine zone with low nutrient levels and dissolved organic materials. Therefore, only a few species from the genera such as *Microcystis*, *Anabaena*, and *Aphanizomenon* in the Cyanophyta phylum, which are adapted to eutrophic water with relatively high water temperature, and abundant dissolved organic substances were observed in the current study (Reynolds et al., 2002; Silva and Costa, 2015). However, temperature was significantly positively correlated with numbers of Cyanophyta and Chlorophyta species. Phytoplankton community composition in the cascade reservoirs in the upstream Yellow River were similar to those reported in the upstream Yangtze River (Chen et al., 2013; Yin et al., 2017), upstream Lancang–Mekong River (Yin et al., 2017), and Qaidam River (Chen et al., 2012) in the Qinghai–Tibet plateau. These regions show characteristics of phytoplankton composition in oligotrophic waters in the alpine region of the Qinghai–Tibet plateau. The finding showed that diatoms had stronger ability to adapt to harsh environments. Nutrient-rich diatoms provide abundant food for aquatic animals in harsh alpine waters and have important ecological significance in the food chain of oligotrophic and alpine ecosystems.

Abundance (1.8 × 10^5^ cells/L) and biovolume (66.6 μg/L) of phytoplankton species in the alpine cascade reservoirs were low. This may be because the cascade reservoirs in the upstream are located on the Qinghai–Tibet plateau, where growth and reproduction of phytoplankton are significantly inhibited by high altitude, low water temperature, and low phosphorus levels (Amaral et al., 2020). Oligotrophic aquatic systems with low species number and biovolume of phytoplankton and simple ecosystem structure are vulnerable to external factors. Low phytoplankton species number, abundance, biovolume, and diversity index observed in the current study indicate that the phytoplankton community in the alpine cascade reservoirs was less stable and less resistant to external disturbance. The findings indicated that the ecosystem of high-altitude rivers is more fragile compared with rivers in low-altitude areas. Alpine river ecosystems are more vulnerable to environmental changes and human activities; therefore, the environmental impact of human activities on alpine river should be carefully explored.

Although some phytoplankton species were present along cascade reservoirs, the phytoplankton community composition was different along cascade reservoirs. Hydrodynamic characteristics of cascade reservoir systems vary worldwide. However, hydrology characteristics are the main factors that modulate the community structure of phytoplankton (Silva et al., 2005; Beaver et al., 2013; Qu et al., 2018). The current study explored the factors regulating the phytoplankton patterns in cascade reservoirs. The findings of this study showed that the environmental factors controlling species number, abundance, biovolume, and biodiversity of phytoplankton can be different in cascade reservoir systems. The species number and the biodiversity index (including the Shannon–Wiener index and Pielou index) of phytoplankton were mainly modulated by the hydrological regime of cascade reservoirs. Hydrological parameters, such as volume, area, and flow were correlated with the drainage rate of cascade reservoirs. Drainage rate/water residence time, and the sediment content of cascade reservoirs were the most important factors modulating the species number and biodiversity index of phytoplankton. This may be because cascade reservoirs with larger water volume, higher drainage rate, and higher sediment content provide spatial heterogeneity to more phytoplankton species (Okuku et al., 2016; Graco-Roza et al., 2020). The findings showed that the hydrological regime modulated phytoplankton species number, abundance, and biovolume of phytoplankton. However, abundance and biovolume of phytoplankton were significantly modulated by nutrient levels compared with the hydrological regime in cascade reservoirs. Higher nitrogen and phosphorus levels promote growth of phytoplankton. Previous studies report that phytoplankton biomass changes in response to nutrient availability mainly in oligotrophic conditions (Özkan et al., 2016; Wang et al., 2018; Amaral et al., 2020). However, some studies report that bioavailable nitrogen and phosphorus are weakly correlated with phytoplankton biomass (Beaver et al., 2013; Okuku et al., 2016). The relationship between nutrient levels and phytoplankton biomass is more complex in flowing cascade reservoir environments owing to multiple structuring factors (Beaver et al., 2013; Silva et al., 2014; Okuku et al., 2016). Hydrology and phosphorus concentrations were significantly correlated with phytoplankton biomass in 12 tropical hydroelectric reservoirs in Brazil (Rangel et al., 2012; Silva et al., 2014). Drainage rate and N:P ratio showed significant negative effects on phytoplankton abundance and biovolume in cascade reservoirs, which was consistent with findings reported by Silva et al. (2014). This can be attributed to the high drainage rate of the cascade reservoirs, which may cause unstable water environment, increased sediment content, and low transparency, which negatively affect the growth of phytoplankton (Silva and Costa, 2015; Okuku et al., 2016; Chen et al., 2018). Notably, high nutrients levels (COD, TN, and TP) significantly promote growth of phytoplankton. Okuku et al. (2016) reported that water retention time is not positively correlated with phytoplankton abundance in cascade reservoirs, and smaller reservoirs showed higher abundance, despite their significantly shorter retention time. However, the findings of the current study showed that LYG (with larger volume, longer water retention time, and more static water) showed the highest phytoplankton abundance and biomass compared with the other cascade reservoirs. In addition, the drainage rate of cascade reservoirs significantly modulated species composition and biovolume of phytoplankton indicating the role of hydraulic stability to phytoplankton (Beaver et al., 2013; Silva and Costa, 2015). The findings showed that increased residence time, low sediment content, and high levels of nutrients increase phytoplankton abundance and biovolume (Bowes et al., 2016; Rao et al., 2018). Furthermore, the findings indicate that sediment content reduced abundance and biovolume of phytoplankton, and increased the diversity index of phytoplankton in the studied cascade reservoirs.

### Zooplankton Community Variation Regulated by Physicochemical Factors

Although previous studies report that physicochemical factors modulate zooplankton communities and dynamics (Sellami et al., 2010; Chang et al., 2014), only a few studies have explored zooplankton community patterns along cascade reservoirs and the factors that regulate these patterns. The findings showed that the composition of the zooplankton community in the studied cascade reservoir system was simple with a few large zooplankton species. A high-altitude environment with low water temperature, strong ultraviolet, and low abundance of phytoplankton and bacteria are negatively correlated with zooplankton abundance (Silva et al., 2014). Therefore, zooplankton species number, abundance, and biovolume were low in the studied alpine cascade reservoir system. High flushing environments in reservoirs favored small-sized zooplankton with short generation times, such as rotifers (Obertegger et al., 2007; Beaver et al., 2013; Silva et al., 2014). Rotifers were dominant in riverine ecosystems with low water residence time (Baranyi et al., 2002; Okuku et al., 2016), which is consistent with the findings of the current study in the flowing cascade reservoir system. However, the findings showed that the species number of rotifers was significantly correlated with the volume and the area of reservoirs, and more species number of Rotatoria was detected in low-flushing LYG compared with other high-flushing reservoirs in our study.

The findings in this 5-year study showed the importance of physicochemical factors on zooplankton communities (Anneville et al., 2007; Sellami et al., 2010; Chang et al., 2014). However, the findings showed that effects of physicochemical factors on species composition, abundance/biovolume and biodiversity index was different in the cascade reservoir system. Zooplankton species number along cascade reservoirs was mainly regulated by the hydrological regime of cascade reservoirs. However, the abundance, biovolume, and biodiversity of zooplankton showed a weak correlation with hydrological regime, and significantly positive correlation with temperature and nutrient levels (TN, TP, and COD). The finding on weak correlation between zooplankton abundance and the hydrology was not consistent with findings from previous studies that high zooplankton abundance was observed in the lentic zones or reservoirs due to reduced water speed and longer retention times (Thorp et al., 1994; Basu and Pick, 1997; Reckendorfer et al., 1999; Okuku et al., 2016). The correlation between abundance and biovolume of zooplankton and temperature, and nutrient levels was because higher temperature and nutrient levels contribute to the growth of phytoplankton through the supply of food for zooplankton (Lampert et al., 1986; Vanni, 1987; Elser and Goldman, 1991; Tavares et al., 2019). Therefore, factors affecting abundance and biovolume of phytoplankton may also affect zooplankton abundance and biovolume. Furthermore, the findings showed that the sediment content did not significantly decrease the Shannon–Wiener index of zooplankton; however, it was significantly correlated with a high Pielou index of zooplankton. A previous study reported that turbidity decreased zooplankton taxa numbers in three tropical cascading reservoirs, which is consistent with the findings of the current study (Okuku et al., 2016).

### Comparison of Effects of Physicochemical Factors on Zooplankton and Phytoplankton Community

Sampling and analysis of both zooplankton and phytoplankton were conducted in this 5-year study. A comparison of the effects of the physicochemical factors on zooplankton and phytoplankton communities showed that the effects of physicochemical factors on zooplankton and phytoplankton showed differences in the cascade reservoir system. Species numbers of phytoplankton and zooplankton along cascade reservoirs were mainly regulated by hydrological regime of cascade reservoirs. This finding indicates the importance of hydrological characteristics in shaping zooplankton and phytoplankton community species composition. However, factors regulating abundance, biovolume, and biodiversity of zooplankton were different from those regulating phytoplankton. Sediment content decreased the Shannon–Wiener index of both phytoplankton and zooplankton in the current study. However, sediment content decreased the Pielou index of phytoplankton but increased the Pielou index of zooplankton. A significant positive correlation between phytoplankton Shannon–Wiener index (*H’*) and Pielou index (*J*) was observed in the present study, which was consistent with findings from previous studies (Stirling and Wilsey, 2001; Ricotta and Avena, 2003; Okuku et al., 2016). This finding indicates that the phytoplankton Shannon–Wiener index (*H’*) was highly correlated with the Pielou index (*J*) compared with phytoplankton abundance and biomass (Okuku et al., 2016). However, insignificant positive correlation was observed between the zooplankton Shannon–Wiener index (*H’*) and the Pielou index (*J*).

### Interaction Between Zooplankton and Phytoplankton Communities

Previous limited numbers of studies on plankton community in cascade reservoir systems only focused on either phytoplankton or zooplankton and seldom focused on both in one study. The findings of the current study showed a significant correlation between phytoplankton and zooplankton in species number, abundance, and biovolume in cascade reservoirs. The significant correlation between zooplankton and phytoplankton species number/composition was consistent with findings from a study by Chang et al. (2014) that explored a subtropical reservoir. Furthermore, a significant correlation was observed between zooplankton and phytoplankton biomass. Strong zooplankton–phytoplankton interactions were expected since zooplankton feeds on phytoplankton (Matveev, 2003; Hunt and Matveev, 2005; Bonecker et al., 2007). However, weak zooplankton–phytoplankton interactions based on biomass have been reported in previous studies (Crisman and Beaver, 1990; Havens et al., 2000; Wang et al., 2007; Yuan and Pollard, 2018). Lack of a detailed investigation on environmental variables–phytoplankton–zooplankton–fish characteristics is partly the reason for these contradicting findings (Chang et al., 2014). Zooplankton biomass is regulated by physicochemical factors, availability of phytoplankton, and fish predation (Yoshida et al., 2001; Hansson et al., 2007; Attayde et al., 2010). The significant correlation between zooplankton and phytoplankton biomass in the studied cascade reservoir can be attributed to a strong bottom–up regulation of phytoplankton (Yuan and Pollard, 2018) and low top–down control of planktivorous fish (Lu and Xie, 2001; Pinto-Coelho et al., 2005; Hansson et al., 2007; Havens et al., 2009; Chang et al., 2014). In the current study, the predation pressure by fish on zooplankton was explored through fish survey. The catches of the current survey and previous studies indicated a low top–down control. Previous studies report low fish species number and fish biomass with very limited planktivorous fishes in alpine cascade reservoirs (Tang and He, 2013; Wang H. et al., 2016; Qiu et al., 2019). Analysis of the biomass, abundance, and distribution of the catches showed that *Hypomesus olidus*, *Gymnocypris eckloni* Herzensten, *Triplophysa scleroptera*, and *Schizopygopsis pylzovi* Kessler were the most dominant species (Tang and He, 2013; Qiu et al., 2019). The dominant taxa, including Schizothorax and Triplophysa in the studied cascade reservoir system, were mainly omnivorous fish. *Schizopygopsis pylzovi* Kessler mainly feed on algae, *Hypomesus olidus* and *Gymnocypris eckloni* Herzensten mainly feed on zooplankton, whereas *Triplophysa scleroptera* mainly feed on benthic gammarid and aquatic insects (Tang and He, 2013). The findings did not show other planktivorous invertebrate predators apart from fish. Therefore, zooplankton biomass was not significantly regulated by fish predation in the studied alpine and oligotrophic cascade reservoirs, which may be different from those in temperate, subtropical, and tropical lakes (Yoshida et al., 2001). Although the top–down control of fish on the zooplankton biomass was low, the findings of the current study showed clear bottom–up regulation of phytoplankton species number, abundance, and biomass on zooplankton community. These findings provide an understanding on interactions between phytoplankton and zooplankton in cascade reservoir system.

### Environmental Implications for Water Conservancy Facilities

The findings of the current study showed that the community structure of plankton is significantly affected by the hydrological regime of the cascade reservoir system (Figures 5, 6). Therefore, plankton community structure is an ideal biomarker to reflect the impact of cascade reservoir construction on the river ecosystem (Li et al., 2013; Graco-Roza et al., 2021). Taxa composition of phytoplankton in the cascade reservoir system were mainly modulated by hydrological conditions (Figures 5, 6). On the contrary, abundance and biomass of phytoplankton were mainly modulated by hydrological conditions and nutrient levels (Figures 5, 6). Drainage rate, sediment content, and nutrient conditions contributed differently in regulating composition, abundance, biovolume, and biodiversity index of both phytoplankton and zooplankton. The hydrological regime can affect material circulation and energy transfer of the cascade reservoir ecosystem by affecting the taxa composition and biomass of plankton (Bertrand et al., 2001; Dias et al., 2016). Therefore, it is the most important regulating factor of the cascade reservoir ecosystem (Figures 5, 6; Li et al., 2013). The findings of the current study can be used to predict the effects of the proposed water conservancy facilities on community composition of plankton. More Chlorophyta species can be expected in a stable environment with low flow velocity and low sediment content, and more diatom species can be expected to survive in the environment of high flow velocity, high sediment content, and low water temperature. Relatively high phytoplankton biomass and abundance can be expected in cascade reservoirs with stable water environment of slow water flow, high transparency, and warm water temperature (Figures 5, 6). Phytoplankton grow and reproduce rapidly and can quickly adapt to changing hydrological characteristics of the cascade reservoir system and form a new plankton community (Wang et al., 2012; Chen et al., 2020). However, benthonic invertebrates with weak mobility and fish, mainly the spawning sites and feeding sites, are much slower to adapt to hydrological changes (Moreno and Callisto, 2006; Zhang et al., 2010). Studies should explore the impact of changes in the structure of plankton community on the community structure of benthonic invertebrates and fish in the upper reaches of rivers. The discharge flow of the reservoir should be kept stable when constructing and operating cascade reservoirs in the upper reaches of the river.

## Conclusion

The longitudinal distribution pattern of plankton and the corresponding regulation factors were systematically explored for the first time in a high-altitude alpine cascade reservoir system. Results showed that plankton community structure is sensitive to the hydrological regime and is an ideal biomarker for evaluating the effects of cascade reservoir construction on a river ecosystem. The findings of the current study also showed that Bacillariophyta and Chlorophyta were the predominant phytoplankton phyla, whereas predominant zooplankton phyla in the alpine and oligotrophic cascade reservoir system were Rotatoria and Protozoa. The phytoplankton and zooplankton species number was mainly regulated by hydrological regime. Notably, drainage rate was the most significant factor, whereas nutrient levels did not significantly have an effect on phytoplankton and zooplankton species abundance. Abundance and biovolume of phytoplankton were modulated by hydrological regime and nutrient levels, whereas abundance and biovolume of zooplankton were mainly regulated by nutrient levels and sediment content. The Shannon–Wiener index and Pielou index of phytoplankton was mainly regulated by drainage rate and sediment content. On the contrary, the Shannon–Wiener index and Pielou index of zooplankton were mainly regulated by temperature and nutrient levels and less regulated by hydrological regime. Sediment content was negatively correlated with abundance and biovolume of both phytoplankton and zooplankton. However, sediment content was positively correlated with the Shannon–Wiener index and Pielou index of phytoplankton, but was negatively correlated with the Shannon–Wiener index of zooplankton. Phytoplankton can quickly adapt to changing hydrological conditions in a cascade reservoir system, and attention should be paid to the changes in community structure of benthonic invertebrates and fish.

## Data Availability Statement

The raw data supporting the conclusions of this article will be made available by the authors, without undue reservation.

## Author Contributions

SJ, YL, and CZL designed the study. KL, SJ, HG, and SM collected and analyzed the samples. YL and CGL drew the figures. YL, CGL, and SJ wrote the manuscript. All authors analyzed and interpreted the data, and approved the final version of the manuscript.

## Conflict of Interest

The authors declare that the research was conducted in the absence of any commercial or financial relationships that could be construed as a potential conflict of interest.

## Publisher’s Note

All claims expressed in this article are solely those of the authors and do not necessarily represent those of their affiliated organizations, or those of the publisher, the editors and the reviewers. Any product that may be evaluated in this article, or claim that may be made by its manufacturer, is not guaranteed or endorsed by the publisher.
